# Large deviation principle for the mean reflected stochastic differential equation with jumps

**DOI:** 10.1186/s13660-018-1889-2

**Published:** 2018-10-26

**Authors:** Yumeng Li

**Affiliations:** School of Statistics and Mathematics, Zhongnan University of Economics and Law, Wuhan, P.R. China

**Keywords:** 60H10, 60H15, Mean reflected stochastic differential equation, Large deviation principle, Weak convergence method

## Abstract

In this paper, we establish a large deviation principle for a mean reflected stochastic differential equation driven by both Brownian motion and Poisson random measure. The weak convergence method plays an important role.

## Introduction

Consider the mean reflected stochastic differential equation (MR-SDE for short) described by the following system:

1 $$ \textstyle\begin{cases} X_{t}=X_{0}+\int_{0}^{t} b(X_{s-})\,ds+\int_{0}^{t} \sigma (X_{s-})\,dB _{s}+\int_{0}^{t}\int_{E} F(X_{s-},z)\widetilde{N}(ds,dz)+K_{t}, \\ \mathbb {E}[h(X_{t})]\ge 0, \quad\quad \int_{0}^{t} \mathbb {E}[h(X_{s})]\,dK_{s}=0, \quad t\ge 0, \end{cases} $$

where $E=\mathbb {R}\setminus \{0\}$, *b*, *σ*, and *F* are Lipschitz functions from $\mathbb {R}$ to $\mathbb {R}$, *h* is bi-Lipschitz continuous, *Ñ* is a compensated Poisson measure $\widetilde{N}(ds,dz)= N(ds,dz)-\vartheta (dz)\,ds$, and $\{B_{t}\}_{t\ge 0}$ is a standard Brownian motion independent of *N*. The integral of the function *h* with respect to the law of the solution to the SDE is asked to be nonnegative. The solution to (1) is the couple of continuous processes $(X,K)$, where *K* is needed to ensure that the constraint is satisfied in a minimal way according to the last condition, namely the Skorokhod condition.

MR-SDE is a very special type of reflected stochastic differentials equations (SDEs) in which the constraint is not directly on the paths of the solution to the SDE as in the usual case but on the law of the solution. This kind of processes has been introduced recently by Briand, Elie, and Hu [4] in backward forms under the context of risk measures. Briand et al. [3] studied the MR-SDE in forward forms, and they provided an approximation of solution to the MR-SDE with the help of interacting particles systems.

Since the original work of Freidlin and Wentzell [11], the small noise large deviation principles for stochastic (partial) differential equations have been extensively studied in the literature. In this setting, one considers a small parameter multiplying the noise term and is interested in asymptotic probabilities of behavior as the parameter approaches zero. Earlier works on this family of problems relied on approximations and exponential probability estimates, see [2, 9]. Later, Dupuis and Ellis [10] developed a weak convergence approach to the theory of large deviation. This approach is mainly based on some variational representation formula about the Laplace transform of bounded continuous functionals. The weak convergence approach has now been adopted for the study of large deviation problems for stochastic partial differential equations, see [8, 13, 14, 16, 17], etc. It is also used to study the moderate deviation problems for stochastic partial differential equations, see [7, 12, 15].

We will use the weak convergence approach to study the large deviation principle for MR-SDE. Here, a representation formula of *K* plays an important role to overcome the difficulty coming from the fact that the reflection process *K* depends on the law of the position.

The rest of this paper is organized as follows. In Sect. 2, we first give the definition of the solution to Eq. (1), and then we state the main results of this paper. The weak convergence criterion for the large deviation principle is recalled in Sect. 3. In Sect. 4, we shall prove the main result.

## Framework and main results

We consider the following conditions.

### Condition 2.1

(i) *Lipschitz assumption*: *For any* $p>0$, *there exists a constant* $C_{p}>0$ *such that*, *for all* $x,x'\in \mathbb {R}$, *we have*
  $$ \bigl\vert b(x)-b\bigl(x'\bigr) \bigr\vert ^{p}+ \bigl\vert \sigma (x)-\sigma \bigl(x'\bigr) \bigr\vert ^{p}+ \int_{E} \bigl\vert F(x,z)-F\bigl(x',z\bigr) \bigr\vert ^{p} \vartheta (dz)\le C_{p} \bigl\vert x-x' \bigr\vert ^{p}. $$
(ii) *The random variable* $X_{0}$ *is square integrable independent of* $B_{t}$ *and* $N_{t}$.

### Condition 2.2

(i) *The function* $h:\mathbb {R}\rightarrow \mathbb {R}$ *is an increasing function*, *and there exist* $0< m< M$ *such that*
  $$ m \vert x-y \vert \le \bigl\vert h(x)-h(y) \bigr\vert \le M \vert x-y \vert \quad \textit{for all } x,y\in \mathbb {R}. $$
(ii) *The initial condition* $X_{0}$ *satisfies* $\mathbb {E}[h(X_{0})] \ge 0$.

### Definition 2.1

A couple of continuous processes $(X,K)$ is said to be a flat deterministic solution to Eq. (1) if $(X,K)$ satisfies (1) with *K* being a nondecreasing deterministic function such that $K_{0}=0$.

### Theorem 2.3

([5, Theorem 1, Proposition 1])

*Under Conditions *2.1 *and *2.2, *the mean reflected SDE* (1) *has a unique deterministic flat solution* $(X, K)$, *and*

2 $$ K_{t}=\sup_{s\le t}\inf \bigl\{ x\ge 0, \mathbb {E}\bigl[h(x+U_{s})\bigr]\ge 0 \bigr\} , $$

*where* $(U_{t})_{0\le t\le T}$ *is the process defined by*

3 $$ U_{t}=x+ \int_{0}^{t} b(X_{s^{-}})\,ds+ \int_{0}^{t} \sigma (X_{s^{-}})\,dB _{s}+ \int_{0}^{t} \int_{E} F(X_{s^{-}},z)\widetilde{N}(ds,dz). $$

*Moreover*, *for any* $p\ge 2$, *there exists a positive constant* $K_{p}$, *depending on* *T*, *b*, *σ*, *F*, *h*, *such that*

4 $$ \mathbb {E}\Bigl[ \sup_{t\le T} \vert X_{t} \vert ^{p} \Bigr] \le K_{p} \bigl( 1+\mathbb {E}\bigl[ \vert X _{0} \vert ^{p} \bigr] \bigr) . $$

In this paper, we are concerned with the large deviation principle (LDP for short) for MR-SDEs of jump type on $\mathbb {R}$:

5 $$ \textstyle\begin{cases} X_{t}^{\varepsilon }=X_{0}+\int_{0}^{t} b(X^{\varepsilon }_{s-})\,ds+\sqrt{\varepsilon }\int_{0} ^{t} \sigma (X^{\varepsilon }_{s-})\,dB_{s}+\int_{0}^{t}\int_{E} F(X^{\varepsilon }_{s-},z) \widetilde{N}^{\varepsilon ^{-1}}(ds,dz)+K^{\varepsilon }_{t}, \\ \mathbb {E}[h(X^{\varepsilon }_{t})]\ge 0, \quad\quad \int_{0}^{t} \mathbb {E}[h(X^{\varepsilon }_{s})]\,dK^{\varepsilon }_{s}=0, \quad t\ge 0. \end{cases} $$

### Condition 2.4

*The function* *F* *satisfies the following*:

1. *There exists a function* $M_{F}\in L^{1}(\vartheta )\cap L^{2}(\vartheta )$ *such that*, *for any* $(x,z)\in \mathbb {R}\times E$,
  $$ \bigl\vert F(x, z) \bigr\vert ^{2}\le M_{F}(z) \bigl(1+ \vert x \vert ^{2}\bigr); $$
2. *There exists a function* $L_{F}\in L^{1}(\vartheta )\cap L^{2}(\vartheta )$ *such that*, *for any* $(x_{i},z)\in \mathbb {R}\times E$, $i=1,2$,
  $$\begin{aligned} \bigl\vert F(x_{1},z)-F(x_{2},z) \bigr\vert ^{2} \leq L_{F}(z) \vert x_{1}-x_{2} \vert ^{2}. \end{aligned}$$

For any $\delta >0$, define a class of functions

6 $$ \mathcal {H}^{\delta }:= \biggl\{ h:E\rightarrow \mathbb {R}: \int_{\varGamma }\exp \bigl( \delta \bigl\vert h(v) \bigr\vert ^{2}\bigr)\vartheta (dv)< \infty , \forall \varGamma \in \mathcal {B}(E)\text{ with } \vartheta (\varGamma )< \infty \biggr\} . $$

### Condition 2.5

*The functions* $M_{F}$ *and* $L_{F}$ *are in the class* $\mathcal {H}^{\delta }$ *for some* $\delta >0$.

### Remark 2.6

Condition 2.5 implies that, for all $\delta \in (0,\infty )$ and $\varGamma \in \mathcal{B}([0,T]\times {E})$ satisfying $\vartheta_{T}(E)<\infty $,

$$\begin{aligned} \int_{\varGamma }\exp \bigl(\delta \bigl\vert M_{F}(v) \bigr\vert \bigr)\vartheta (dv)\,ds< \infty \quad \text{and} \quad \int_{\varGamma }\exp \bigl(\delta \bigl\vert L_{F}(v) \bigr\vert \bigr)\vartheta (dv)\,ds< \infty . \end{aligned}$$

The main result of this paper is the following theorem.

### Theorem 2.7

*Suppose that Conditions* 2.1, 2.2, 2.4, *and *2.5 *hold*. *Then the family* $\{X^{\varepsilon }\}_{\varepsilon >0}$ *satisfies a large deviation principle on* $D([0,T];\mathbb {R})$ *in the topology of uniform convergence with the rate function* *I* *defined as in* (11) *with* $\mathcal{G}^{0}$ *given by* (22). *More precisely*, *for any Borel set* $\varGamma \in D([0,T];\mathbb {R})$, *we have*

$$ -\inf_{\gamma \in \mathring{\varGamma }}I(\gamma )\le \liminf_{\varepsilon \rightarrow 0} \varepsilon \log \mathbb {P}\bigl(X^{\varepsilon }\in \varGamma \bigr) \limsup_{\varepsilon \rightarrow 0} \varepsilon \log \mathbb {P}\bigl(X^{\varepsilon }\in \varGamma \bigr)\le - \inf_{\gamma \in \overline{\varGamma }}I( \gamma ). $$

## Poisson random measure and the weak convergence criterion

### Poisson random measure and Brownian motion

Let *E* be a locally compact Polish space. Denote by $C([0,T],{E})$ and $D([0,T],{E})$ the spaces of continuous functions and right continuous functions with left limits from $[0,T]$ into *E*, respectively; $C_{c}({E})$ is the space of continuous functions with compact supports, $\mathcal{M}_{FC}({E})$ is the space of all measures *ϑ* on $({E},\mathcal{B}({E}))$ such that $\vartheta (K)<\infty $ for every compact *K* in *E*. Endow $\mathcal{M}_{FC}({E})$ with the weakest topology such that, for every $f\in C_{c}({E})$, the function $\vartheta \rightarrow \langle f,\vartheta \rangle =\int_{{E}}f(u)\,d \vartheta (u)$ is continuous. For any $T\in (0,\infty )$, let ${E}_{T}:=[0,T]\times {E}$. For the measure $\vartheta \in \mathcal{M}_{FC}({E})$, let $\vartheta_{T}:=\lambda_{T}\otimes \vartheta $, where $\lambda_{T}$ is the Lebesgue measure on $[0,T]$.

Recall that a Poisson random measure **n** on ${E}_{T}$ with intensity measure $\vartheta_{T}$ is an $\mathcal{M}_{FC}({E}_{T})$-valued random variable such that, for each $B\in \mathcal{B}({E}_{T})$ with $\vartheta_{T}(B)<\infty $, $\mathbf{n}(B)$ is Poisson distributed with mean $\vartheta_{T}(B)$, and for disjoint $B_{1},\ldots,B_{k}\in \mathcal{B}({E}_{T})$, $\mathbf{n}(B_{1}),\ldots,\mathbf{n}(B_{k})$ are mutually independent random variables. Denote by $\mathbb{P}$ the measure induced by **n** on $(\mathcal{M}_{FC}({E}_{T}),\mathcal{B}(\mathcal{M} _{FC}({E}_{T})))$. Let $\mathbb{M}:=\mathcal{M}_{FC}({E}_{T})$. Then $\mathbb{P}$ is the unique probability measure on $(\mathbb{M}, \mathcal{B}(\mathbb{M}))$ under which the canonical map $N:\mathbb{M} \rightarrow \mathbb{M}$, $N(m):= m$ is a Poisson random measure with intensity measure $\vartheta_{T}$. For each $\theta >0$, let $\mathbb{P}_{\theta }$ be the probability measure on $(\mathbb{M}, \mathcal{B}(\mathbb{M}))$ under which *N* is a Poisson random measure with intensity $\theta \vartheta_{T}$.

Set $\mathbb{Y}:={E}\times [0,\infty )$ and $\mathbb{Y}_{T}:=[0,T] \times \mathbb{Y}$. Similarly, let $\overline{\mathbb{M}}:= \mathcal{M}_{FC}(\mathbb{Y}_{T})$ and $\overline{\mathbb{P}}$ be the unique probability measure on $(\overline{\mathbb{M}},\mathcal{B}(\overline{ \mathbb{M}}))$ under which the canonical map $\overline{N}:\overline{ \mathbb{M}}\rightarrow \overline{\mathbb{M}}$, $\overline{N}(m):= m$ is a Poisson random measure with intensity measure $\overline{\vartheta } _{T}:=\lambda_{T}\otimes \vartheta \otimes \lambda_{\infty }$, with $\lambda_{\infty }$ being Lebesgue measure on $[0,\infty )$. Let $\mathcal{F}_{t}:=\sigma \{\overline{N}((0,s]\times O):0\leq s\leq t, O\in \mathcal{B}(\mathbb{Y})\}$, and denote by $\overline{\mathcal{F}}_{t}$ the completion under $\overline{ \mathbb{P}}$. Set $\overline{\mathcal{P}}$ to be the predictable *σ*-field on $[0,T]\times \overline{\mathbb{M}}$ with the filtration $\{\overline{\mathcal{F}}_{t}:0\leq t\leq T\}$ on $(\overline{\mathbb{M}},\mathcal{B}(\overline{\mathbb{M}}))$. Let $\overline{\mathcal{A}}$ be the class of all $(\overline{\mathcal{P}} \otimes \mathcal{B}({E}))/\mathcal{B}[0,\infty )$-measurable maps $\varphi :{E}_{T}\times \overline{\mathbb{M}}\rightarrow [0,\infty )$. For $\varphi \in \overline{\mathcal{A}}$, define a counting process $N^{\varphi }$ on ${E}_{T}$ by

7 $$\begin{aligned} N^{\varphi }((0,t]\times U):= \int_{(0,t]\times U} \int_{(0,\infty )}1_{[0, \varphi (s,x)]}(r)\overline{N}(\,ds\,dx\,dr), \quad t \in [0,T],U\in \mathcal{B}( {E}). \end{aligned}$$

$N^{\varphi }$ is the controlled random measure with *φ* selecting the intensity for the points at location *x* and time *s*.

Set $\mathbb{W}:=C([0,T],\mathbb{R} )$, $\mathbb{V}:=\mathbb{W}\times \mathbb{M}$, and $\overline{\mathbb{V}}:=\mathbb{W}\times \overline{ \mathbb{M}}$. Define $N^{\mathbb{V}}: \mathbb{V}\rightarrow \mathbb{M}$ as $N^{\mathbb{V}}(w,m)=m$ for $(w,m)\in \mathbb{V}$, and $B^{\mathbb{V}}$ by $\beta^{\mathbb{V}}(w,m)=w$ for $(w,m)\in \mathbb{V}$. The maps $\overline{N}^{\overline{\mathbb{V}}}: \overline{ \mathbb{V}}\rightarrow \overline{\mathbb{M}}$ and $B^{\overline{ \mathbb{V}}}=(B^{\overline{\mathbb{V}}}$ are defined analogously. Define the *σ*-filtration $\mathcal{G}^{\mathbb{V}}_{t}:=\sigma \{N ^{\mathbb{V}}((0,s]\times O),B^{\mathbb{V}}(s): 0\leq s\leq t, O \in \mathcal{B}({E})\}$. For every $\theta >0$, $\mathbb{P}^{ \mathbb{V}}_{\theta }$ denotes the unique probability measure on $(\mathbb{V},\mathcal{B}(\mathbb{V}))$ such that:

1. $B^{\mathbb{V}}$ is a standard Brownian motion,
2. $N^{\mathbb{V}}$ is a Poisson random measure with intensity measure $\theta \vartheta_{T}$ independent of $B^{\mathbb{V}}$.

Analogously, define $( \overline{\mathbb{P}}^{\overline{ \mathbb{V}}}_{\theta },\overline{\mathcal{G}}^{\overline{\mathbb{V}}} _{t} ) $ and denote $\overline{\mathbb{P}}^{\overline{\mathbb{V}}} _{\theta =1}$ by $\overline{\mathbb{P}}^{\overline{\mathbb{V}}}$. Denote by $\{ \overline{\mathcal{F}}^{\overline{\mathbb{V}}}_{t} \} $ the $\overline{\mathbb{P}}^{\overline{\mathbb{V}}}$-completion of $\{ \overline{\mathcal{G}}^{\overline{\mathbb{V}}}_{t} \} $ and by $\overline{\mathcal{P}}^{\overline{\mathbb{V}}}$ the predictable *σ*-field on $[0,T]\times \overline{\mathbb{V}}$ with the filtration $\{\overline{\mathcal{F}}^{\overline{\mathbb{V}}}_{t}\}$ on $(\overline{\mathbb{V}},\mathcal{B}(\overline{\mathbb{V}}))$. Let $\overline{\mathbb{A}}$ be the class of all $( \overline{ \mathcal{P}}^{\overline{\mathbb{V}}}\otimes \mathcal{B}({E}) ) / \mathcal{B}[0,\infty )$-measurable maps $\varphi :{E}_{T}\times \overline{ \mathbb{V}}\rightarrow [0,\infty )$. Define $l:[0,\infty )\rightarrow [0,\infty )$ by

$$ l(r):=r\log r-r+1,\quad r\in [0,\infty ). $$

For any $\varphi \in \overline{\mathbb{A}}$, the quantity

8 $$\begin{aligned} L_{T}(\varphi ):= \int_{{E}_{T}}l\bigl(\varphi (t,x,\omega )\bigr) \vartheta_{T}(\,dt\,dx) \end{aligned}$$

is well defined as a $[0,\infty ]$-valued random variable.

Let

9 $$\begin{aligned} \mathcal{L}_{2}:= \biggl\{ \psi : \psi \text{ is } \overline{ \mathcal{P}}^{\overline{\mathbb{V}}}\setminus \mathcal{B}(\mathbb{R}) \text{ measurable and } \int_{0}^{T} \bigl\vert \psi (s) \bigr\vert ^{2}\,ds< \infty ,\text{a.s.-} \overline{\mathbb{P}}^{\overline{\mathbb{V}}} \biggr\} . \end{aligned}$$

Set $\mathcal{U}:=\mathcal{L}_{2}\times \overline{\mathbb{A}}$. Define

$$ \tilde{L}_{T}(\psi ):=\frac{1}{2} \int_{0}^{T} \bigl\vert \psi (s) \bigr\vert ^{2} \,ds, \quad \forall \psi \in \mathcal{L}_{2}, $$

and

10 $$ \overline{L}_{T}(u):=\tilde{L}_{T}(\psi )+L_{T}(\varphi ), \quad \forall u=(\psi ,\varphi )\in \mathcal{U}. $$

### The weak convergence criterion

In this subsection, we recall a general criterion for a large deviation principle established in [8]. Let $\{\mathcal{G}^{\varepsilon }\}_{\varepsilon >0}$ be a family of measurable maps from $\overline{\mathbb{V}}$ to $\mathbb{U}$, where $\overline{\mathbb{V}}$ is introduced in Sect. 3.1 and $\mathbb{U}$ is a Polish space. We present a sufficient condition of large deviation principle for the family $Z^{\varepsilon }:=\mathcal{G}^{\varepsilon } ( \sqrt{\varepsilon }B, \varepsilon N^{\varepsilon^{-1}} ) $, as $\varepsilon \rightarrow 0$.

Define

$$\begin{aligned} S^{\varUpsilon }:= \bigl\{ g:E_{T}\rightarrow [0,\infty );L_{T}(g)\leq {\varUpsilon } \bigr\} \end{aligned}$$

and

$$\begin{aligned} \tilde{S}^{\varUpsilon }:= \bigl\{ f:L^{2}\bigl([0,T],\mathbb {R}\bigr); \tilde{L}_{T}(f) \leq {\varUpsilon } \bigr\} . \end{aligned}$$

A function $g\in S^{\varUpsilon }$ can be identified with a measure $\vartheta_{T}^{g}\in \mathbb{M}$ defined by

$$\begin{aligned} \vartheta_{T}^{g}(O):= \int_{O} g(s,x)\vartheta_{T}(\,ds\,dx), \quad O\in \mathcal{B}(E_{T}). \end{aligned}$$

This identification induces a topology on $S^{\varUpsilon }$ under which $S^{\varUpsilon }$ is a compact space, see the Appendix of [6]. Throughout we use this topology on $S^{\varUpsilon }$. We also use the weak topology on $\tilde{S}^{\varUpsilon }$. Set $\overline{S}^{{\varUpsilon }}:=\tilde{S}^{{\varUpsilon }}\times S ^{{\varUpsilon }}$, $\mathbb{S}:=\bigcup_{{\varUpsilon }\geq 1}\overline{S} ^{\varUpsilon }$, and

$$ \mathcal{U}^{\varUpsilon }:= \bigl\{ u=(\psi ,\varphi )\in \mathcal{U}: u( \omega )\in \overline{S}^{\varUpsilon },\overline{\mathbb{P}}^{\overline{ \mathbb{V}}} \text{-a.e. }\omega \bigr\} . $$

The following condition is sufficient for establishing an LDP for a family $\{Z^{\varepsilon }\}_{\varepsilon >0}$.

#### Condition 3.1

*There exists a measurable map* $\mathcal{G}^{0}:\overline{\mathbb{V}} \rightarrow \mathbb{U}$ *such that the following conditions hold*:

- *For any* ${\varUpsilon }\in \mathbb{N}$, *let* $(f_{n},g_{n}), (f,g) \in \overline{S}^{\varUpsilon }$ *be such that* $(f_{n},g_{n})\rightarrow (f,g)$ *as* $n\rightarrow \infty $. *Then*
  $$ \mathcal{G}^{0} \biggl( \int_{0}^{\cdot }f_{n}(s)\,ds, \vartheta_{T}^{g _{n}} \biggr) \longrightarrow \mathcal{G}^{0} \biggl( \int_{0}^{\cdot }f(s)\,ds, \vartheta_{T}^{g} \biggr) \quad \textit{in } \mathbb{U}. $$
- *For any* ${\varUpsilon }\in \mathbb{N}$, *let* $\phi_{\varepsilon }= ( \psi_{\varepsilon },\varphi_{\varepsilon } ) $, $\phi =( \psi ,\varphi )\in \mathcal{U}^{\varUpsilon }$ *be such that* $\phi_{\varepsilon }$ *converges in distribution to* *ϕ* *as* $\varepsilon \rightarrow 0$. *Then*
  $$ \mathcal{G}^{\varepsilon } \biggl( \sqrt{\varepsilon }B+ \int_{0}^{ \cdot }\psi_{\varepsilon }(s)\,ds, \varepsilon N^{\varepsilon^{-1} \varphi_{\varepsilon }} \biggr) \longrightarrow \mathcal{G}^{0} \biggl( \int _{0}^{\cdot }\psi (s)\,ds,\vartheta_{T}^{\varphi } \biggr) \quad \textit{in distribution}. $$

For $h\in \mathbb{U}$, define $\mathbb{S}_{h}:= \{ (f,g)\in \mathbb{S}:h=\mathcal{G}^{0} ( \int_{0}^{\cdot }f(s)\,ds, \vartheta ^{g}_{T} ) \} $. Let $I:\mathbb{U}\rightarrow [0,\infty ]$ be defined by

11 $$\begin{aligned} I(h):=\inf_{q=(f,g)\in \mathbb{S}_{h}} \bigl\{ \overline{L}_{T}(q) \bigr\} , \quad h\in \mathbb{U}, \end{aligned}$$

where $\overline{L}_{T}(q)$ is given by (10). By convention, $I(h)=\infty $ if $\mathbb{S}_{h}=\emptyset $.

Recall the following criterion from [8].

#### Theorem 3.2

([8])

*Suppose that Condition *3.1 *holds*. *Then the family* $\{ \mathcal{G} ^{\varepsilon } ( \sqrt{\varepsilon }B,\varepsilon N^{ \varepsilon^{-1}} ) \} _{\varepsilon >0}$ *satisfies a large deviation principle with the rate function* *I* *given by* (11).

For applications, the following strengthened form of Theorem 3.2 is useful. Let $\{K_{n}\}_{n\ge 1}$ be an increasing sequence of compact sets in $\mathbb{X}$ such that $\bigcup_{n=1}^{ \infty }K_{n}={E}$. For each *n*, let

$$\begin{aligned} \overline{\mathbb{A}}_{b,n} := \bigl\{ \varphi \in \overline{ \mathbb{A}}: 1/n \leq \varphi (\cdot ,x,\cdot )\leq n \text{ if } x \in K_{n}; \varphi (\cdot ,x,\cdot )=1 \text{ if } x\in K_{n} ^{c} \bigr\} , \end{aligned}$$

and let $\overline{\mathbb{A}}_{b}:=\bigcup_{n=1}^{\infty }\overline{ \mathbb{A}}_{b,n}$, $\tilde{\mathcal{U}}^{\varUpsilon }:=\mathcal{U}^{ \varUpsilon }\cap \{ (\psi ,\phi ): \phi \in \overline{\mathbb{A}}_{b} \} $.

#### Theorem 3.3

([8])

*Suppose Condition *3.1 *holds with* $\mathcal{U}^{\varUpsilon }$ *replaced by* $\tilde{\mathcal{U}}^{\varUpsilon }$. *Then the conclusions of Theorem *3.2 *also hold*.

## Proof of Theorem 2.7

### Proof of Theorem 2.7

According to Theorem 3.3, we need to prove that Condition 3.1 is fulfilled. The verification of Conditions (3.1.a) and (3.1.b) will be given by Proposition 4.4 and Proposition 4.8, respectively. □

To use the representation formula (2) of the process *K*, we recall a result from [3]. Define the function

$$ H:\mathbb {R}\times \mathcal{P}(\mathbb {R})\ni (x,\nu )\longmapsto H(x,v)= \int h(x+z) \nu (dz), $$

and

12 $$ G_{0}:\mathcal{P}(\mathbb {R})\ni \nu \longmapsto \inf \bigl\{ x\ge 0: H(x,v) \ge 0\bigr\} . $$

With these notations, denoting by $(\mu_{t})_{t\in [0,T]}$ the family of marginal laws of $(U_{t})_{t\in [0,T]}$, we have

13 $$ K_{t}=\sup_{s\le t}G_{0}( \mu_{s}). $$

For any two measures *ν* and $\nu '$, the Wasserstein-1 distance between *ν* and $\nu '$ is defined by

$$ W_{1}\bigl(\nu ,\nu '\bigr)= \inf_{X\sim \nu , Y\sim \nu '} \mathbb {E}\bigl[ \vert X-Y \vert \bigr] . $$

### Lemma 4.1

([3, Theorem 2.5])

*Under* (A.2), *for any* $\nu , \nu '\in \mathcal{P}(\mathbb {R})$,

$$ \bigl\vert G_{0}(\nu )-G_{0}\bigl(\nu ' \bigr) \bigr\vert \le \frac{M}{m} W_{1}\bigl(\nu ,\nu '\bigr). $$

From Remark 1 in [5], we have

14 $$ K_{t}-K_{s}=\sup_{s\le r\le t}G_{0}(X_{s}+U_{r}-U_{s}). $$

By the definition of $G_{0}(X_{s})=0$, if $s< t$, using Lemma 4.1, we have

15 $$\begin{aligned} \vert K_{t}-K_{s} \vert = {}&\sup_{s\le r\le t}G_{0}(X_{s}+U_{r}-U_{s}) \\ ={} &\sup_{s\le r\le t} \bigl\vert G_{0}(X_{s}+U_{r}-U_{s})-G_{0}(X_{s}) \bigr\vert \\ \le {}& \frac{M}{m}\sup_{s\le r\le t}\mathbb {E}\bigl[ \vert U_{r}-U_{s} \vert \bigr] . \end{aligned}$$

The following lemma can be proved by using the argument in [6, Lemma 3.4], [7, Lemma 4.3]. We omit its proof.

### Lemma 4.2

*Under Conditions *2.4 *and *2.5, *for the function* $G=M_{F}$ *or* $L_{F}$, *we have*

(i) *For every* ${\varUpsilon }\in \mathbb{N}$,
  16 $$\begin{aligned} C^{\varUpsilon }_{1}:=\sup_{g\in S^{\varUpsilon }} \int_{{E}_{T}} \bigl\vert G(v) \bigr\vert \cdot \bigl\vert g(s,v)-1 \bigr\vert \vartheta (dv)\,ds< \infty \end{aligned}$$
  *and*
  17 $$\begin{aligned} C^{\varUpsilon }_{2}:=\sup_{g\in S^{\varUpsilon }} \int_{{E}_{T}} \bigl\vert G(v) \bigr\vert ^{2} \cdot \bigl(g(s,v)+1\bigr)\vartheta (dv)\,ds< \infty . \end{aligned}$$
(ii) *For ever* $\eta >0$, *there exists* $\delta >0$ *such that*, *for any* $A\subset [0,T]$ *satisfying* $\lambda_{T}(A)<\delta $,
  18 $$\begin{aligned} \sup_{g\in S^{\varUpsilon }} \int_{A} \int_{{E}} \bigl\vert G(v) \bigr\vert \cdot \bigl\vert g(s,v)-1 \bigr\vert \vartheta (dv)\,ds\leq \eta . \end{aligned}$$

The following lemma is from [6, Lemma 3.11].

### Lemma 4.3

*Let* $k:[0,T]\times E\rightarrow \mathbb{R}$ *be a measurable function such that*

$$\begin{aligned} \int_{{E}_{T}} \bigl\vert k(s,v) \bigr\vert ^{2} \vartheta (dv)\,ds< \infty , \end{aligned}$$

*and for all* $\delta \in (0,\infty )$ *and* $\varGamma \in \mathcal{B}([0,T] \times E)$ *satisfying* $\vartheta_{T}(\varGamma )<\infty $,

$$\begin{aligned} \int_{\varGamma }\exp \bigl( \delta \bigl\vert k(s,v) \bigr\vert \bigr) \vartheta (dv)\,ds< \infty . \end{aligned}$$

*For any* ${\varUpsilon }\in \mathbb{N}$, *let* $g_{n},g\in S^{\varUpsilon }$ *be such that* $g_{n}\rightarrow g$ *as* $n\rightarrow \infty $. *Then*

$$\begin{aligned} \lim_{n\rightarrow \infty } \int_{E_{T}}k(s,v) \bigl(g_{n}(s,v)-1\bigr)\vartheta (dv)\,ds= \int_{E_{T}}k(s,v) \bigl(g(s,v)-1\bigr)\vartheta (dv)\,ds. \end{aligned}$$

Under Conditions (A.1) and (A.2), Eq. (5) has a unique strong solution $X^{\varepsilon }$. Therefore, there exists a Borel-measurable function $\mathcal{G}^{\varepsilon }: \bar{\mathbb{V}}\rightarrow D([0,T]; \mathbb {R})$ such that, for any Poisson random measure $N^{\varepsilon^{-1}}$ on $[0,T]\times E$ with intensity measure $\varepsilon^{-1} \lambda_{T} \otimes \vartheta $, $\mathcal{G}^{\varepsilon } ( \sqrt{\varepsilon }B, \varepsilon N^{\varepsilon^{-1}} ) $ is the unique solution of Eq. (5).

Next we introduce the map $\mathcal{G}^{0}$ which will be used to define the rate function and also used for verification of Condition 3.1. Recall $\mathbb{S}$ defined in the last section. Under Condition 2.4, for every $q=(f,g)\in \mathbb{S}$, the deterministic integral equation

19 $$ \textstyle\begin{cases} X_{t}^{q}=X_{0}+\int_{0}^{t} b(X^{q}_{s})\,ds+\int_{0}^{t} \sigma (X ^{q}_{s})f(s)\,ds \\ \hphantom{X_{t}^{q}=}{} +\int_{0}^{t}\int_{E} F ( X^{q}_{s}, v ) ( g(s,v)-1 ) \vartheta (dz)\,ds+K^{q}_{t}, \\ \mathbb {E}[h(X^{q}_{t})]\ge 0, \quad\quad \int_{0}^{t} h(X^{q}_{s})\,dK^{q}_{s}=0, \quad t\ge 0, \end{cases} $$

has a unique continuous solution. Here

20 $$ K_{t}^{q}=\sup_{s\le t}\inf \bigl\{ x\ge 0, h\bigl(x+U^{q}_{s}\bigr)\ge 0\bigr\} , $$

where $(U_{t})_{0\le t\le T}$ is the process defined by

21 $$\begin{aligned} U_{t}^{q}= {}&X_{0}+ \int_{0}^{t} b\bigl(X^{q}_{s} \bigr)\,ds+ \int_{0}^{t} \sigma \bigl(X ^{q}_{s} \bigr)f(s)\,ds+ \int_{0}^{t} \int_{E} F \bigl( X^{q}_{s}, v \bigr) \bigl( g(s,v)-1 \bigr) \vartheta (dz)\,ds. \end{aligned}$$

Define

22 $$\begin{aligned} \mathcal{G}^{0} \biggl( \int_{0}^{\cdot }f(s)\,ds,\vartheta^{g}_{T} \biggr) := {X}^{q}\quad \text{for } q=(f,g)\in \mathbb{S}. \end{aligned}$$

Let $I:D([0,T];\mathbb {R})\rightarrow [0,\infty ]$ be defined as in (11) with $\mathcal{G}^{0}$ given by (22).

We first verify Condition (3.1.a).

### Proposition 4.4

*Let* ${\varUpsilon }\in \mathbb{N}$ *and let* $q_{n}:=(f_{n},g_{n})$, $q:=(f,g) \in \bar{S}^{\varUpsilon }$ *be such that* $q_{n}\rightarrow q$ *as* $n\rightarrow \infty $ *in* $\bar{S}^{\varUpsilon }$. *Then*

$$\begin{aligned} \mathcal{G}^{0} \biggl( \int_{0}^{\cdot }f_{n}(s)\,ds, \vartheta_{T}^{g_{n}} \biggr) \longrightarrow \mathcal{G}^{0} \biggl( \int_{0}^{\cdot }f(s)\,ds,\vartheta _{T}^{g} \biggr) \quad \textit{in } C\bigl([0,T];\mathbb {R}\bigr). \end{aligned}$$

### Proof

Recall that $\mathcal{G}^{0} ( \int_{0}^{\cdot }f_{n}(s)\,ds, \vartheta_{T}^{g_{n}} ) ={X}^{q_{n}}$. For simplicity, we denote $X^{n}:={X}^{q_{n}}$, $X:={X}^{q}$. Since $X\in C([0, T];\mathbb {R})$, we know that $M:=\sup_{t\in [0,T]}\vert X(t)\vert <+\infty $. Notice that

23 $$\begin{aligned} X^{n}_{t}-X_{t}= {} & \int_{0}^{t} \bigl[ b\bigl(X^{n}_{s} \bigr)-b(X_{s}) \bigr] \,ds+ \int _{0}^{t} \bigl[ \sigma \bigl(X^{n}_{s} \bigr)f_{n}(s)-\sigma (X_{s})f(s) \bigr] \,ds \\ & {} + \int_{0}^{t} \int_{\mathbb {X}} \bigl[ G\bigl(X^{n}_{s}, z \bigr) \bigl(g_{n}(s,z)-1\bigr)-G(X _{s}, z) \bigl(g(s,z)-1 \bigr) \bigr] \vartheta (dz)\,ds \\ & {} +K^{q_{n}}_{t}-K^{q}_{t} \\ = {} & \int_{0}^{t} \bigl[ b\bigl(X^{n}_{s} \bigr)-b(X_{s}) \bigr] \,ds+ \int_{0}^{t} \bigl[ \sigma \bigl(X ^{n}_{s}\bigr)f_{n}(s)-\sigma (X_{s})f_{n}(s) \bigr] \,ds \\ & {} + \int_{0}^{t} \bigl[ \sigma (X_{s})f_{n}(s)- \sigma (X_{s})f(s) \bigr] \,ds \\ & {} + \int_{0}^{t} \int_{E} \bigl[ G\bigl(X^{n}_{s}, z \bigr) \bigl(g_{n}(s,z)-1\bigr)-G(X_{s}, z) \bigl(g_{n}(s,v)-1\bigr) \bigr] \vartheta (dv)\,ds \\ & {} + \int_{0}^{t} \int_{E} \bigl[ G(X_{s}, v) \bigl(g_{n}(s,v)-1 \bigr)-G(X_{s}, v) \bigl(g(s,v)-1\bigr) \bigr] \vartheta (dv)\,ds \\ & {} +K^{q_{n}}_{t}-K^{q}_{t} \\ =: {}&I^{n}_{1}(t)+I^{n}_{2}(t)+I^{n}_{3}(t)+I^{n}_{4}(t)+I^{n}_{5}(t)+I ^{n}_{6}(t). \end{aligned}$$

Set $\kappa^{n}(t):=\sup_{u\in [0, t]}\vert X^{n}(u)-X(u)\vert $. By the Lipschitz condition of *b*, we have

24 $$\begin{aligned} \bigl\vert I^{n}_{1}(t) \bigr\vert \le \int_{0}^{t} \bigl\vert b\bigl(X^{n}_{s} \bigr)-b(X_{s}) \bigr\vert \,ds \le C \int_{0}^{t} \kappa^{n}(s) \,ds. \end{aligned}$$

By the Lipschitz condition of *σ*, we have

25 $$\begin{aligned} \bigl\vert I^{n}_{2}(t) \bigr\vert \le {}&C \int_{0}^{t} \kappa^{n}(s)\cdot \bigl\vert f_{n}(s) \bigr\vert \,ds. \end{aligned}$$

By the linear growth of *σ*, we know that

26 $$ \sup_{t\in [0, T]} \bigl\vert \sigma (X_{t}) \bigr\vert ^{2}\le K\bigl(1+2M^{2}\bigr). $$

Since $f_{n}\rightarrow f$ weakly in $L^{2}([0,T];\mathbb {R})$, there exists a constant $C(f)$ such that

$$ \int_{0}^{T} \bigl\vert f_{n}(s) \bigr\vert ^{2}\,ds\le C(f) \quad \text{and}\quad \int_{0}^{T} \bigl\vert f(s) \bigr\vert ^{2}\,ds\le C(f). $$

Thus, by the Cauchy–Schwarz inequality, we have

$$\begin{aligned} \sup_{t\in [0,T]} \bigl\vert I^{n}_{3}(t) \bigr\vert \le {}& \int_{0}^{T} \bigl\vert \sigma (X_{s}) \bigl(f _{n}(s)-f(s)\bigr) \bigr\vert \,ds \\ \le {}& \biggl( \int_{0}^{T} \bigl\vert \sigma (X_{s}) \bigr\vert ^{2}\,ds \biggr) ^{ \frac{1}{2}} \cdot \biggl( \int_{0}^{T} \bigl\vert f_{n}(s)-f(s) \bigr\vert ^{2}\,ds \biggr) ^{\frac{1}{2}} \\ \le {}& \Bigl( T\cdot \sup_{t\in [0, T]} \bigl\vert \sigma ( X_{t}) \bigr\vert ^{2} \Bigr) ^{\frac{1}{2}} \cdot \biggl( 2 \int_{0}^{T}\bigl( \bigl\vert f_{n}(s) \bigr\vert ^{2}+ \bigl\vert f(s) \bigr\vert ^{2}\bigr) \,ds \biggr) ^{\frac{1}{2}} \\ \le {}& \bigl[ CT\bigl(1+2M^{2}\bigr) C(f) \bigr] ^{\frac{1}{2}}< \infty . \end{aligned}$$

Similarly, we have, for any $0\le t_{1}< t_{2}\le T$,

$$\begin{aligned} \bigl\vert I_{3}^{n}(t_{2})-I_{3}^{n}(t_{1}) \bigr\vert \le {}& \bigl[ C(t_{2}-t_{1}) \bigl(1+2M ^{2}\bigr) C(f) \bigr] ^{\frac{1}{2}}, \end{aligned}$$

which means that the sequence $\{I_{3}^{n}:n\ge 1\}$ is equicontinuous. By the Arzéla–Ascoli theorem, we know that $\{I_{3}^{n}:n\ge 1\}$ is relatively compact in $C([0,T];\mathbb {R}^{d})$.

By using (26) and the fact that $f_{n}\rightarrow f$ weakly in $L^{2}([0,T];\mathbb {R})$, we obtain that, for any $t\in [0,T]$,

$$\begin{aligned} I_{3}^{n}(t) &= \int_{0}^{t} \bigl(f_{n}(s)-f(s)\bigr) \sigma (X_{s}) \,ds\longrightarrow 0, \quad \text{as } n\rightarrow \infty . \end{aligned}$$

This, together with the relative compactness of $\{I_{3}^{n}:n\ge 1\}$ in $C([0,T];\mathbb {R})$, implies that

27 $$\begin{aligned} \lim_{n\rightarrow \infty }\sup_{t\in [0,T]} \bigl\vert I_{3}^{n}(t) \bigr\vert =0. \end{aligned}$$

By Lemma 4.2, we have

28 $$\begin{aligned} \bigl\vert I^{n}_{4}(t) \bigr\vert \le {}& 2 \int_{0}^{t} \kappa^{n}(s) \bigl\vert L_{F}(v) \bigr\vert \cdot \bigl\vert g _{n}(s,v)-1 \bigr\vert \,ds. \end{aligned}$$

By Condition 2.4, Remark 2.6, and Lemma 4.3, we know that as $n\rightarrow \infty $,

29 $$\begin{aligned} I^{n}_{5}(t)= \int_{0}^{t} \int_{E}G(X_{s}, v) \bigl[ g_{n}(s,v)-g(s,v) \bigr] \vartheta (dv)\,ds\longrightarrow 0, \quad \forall t\in [0,T]. \end{aligned}$$

By Lemma 4.2, we know that the sequence $\{I^{n}_{5}:n\ge 1\}$ is uniformly bounded and equicontinuous, which implies that $\{I^{n}_{5}:n\ge 1\}$ is relatively compact in $C([0,T];\mathbb {R}^{d})$ by the Arzéla–Ascoli theorem. Consequently, by (29), we know

30 $$\begin{aligned} \lim_{n\rightarrow \infty }\sup_{ t\in [0, T]} \bigl\vert I^{n}_{5}(t) \bigr\vert \rightarrow 0. \end{aligned}$$

Recalling the definition of $K_{t}^{q}$ given by (21), we have

31 $$\begin{aligned} K_{t}^{q_{n}}-K^{q}_{t}=\sup _{s\le t}\inf \bigl\{ x\ge 0, h \bigl( x+U ^{q}_{s} \bigr) \ge 0 \bigr\} . \end{aligned}$$

According to Lemma 4.1, we know that

32 $$\begin{aligned} K_{t}^{q_{n}}-K^{q}_{t}\le {}& \frac{M}{m}\sup_{0\le s\le t} \bigl\vert U^{q_{n}} _{s}-U^{q}_{s} \bigr\vert =\frac{M}{m} \sup_{0\le s\le t}\sum_{i=1}^{5} \bigl\vert I^{n} _{i} \bigr\vert (s). \end{aligned}$$

Putting (23), (24), (25), (28), (32) together, we have

$$\begin{aligned} \kappa^{n}(t)\le {}&C \biggl( \int_{0}^{t} \kappa^{n}(s) \,ds+ \int_{0}^{t} \kappa^{n}(s)\cdot \bigl\vert f_{n}(s) \bigr\vert \,ds+ \int_{0}^{t} \kappa^{n}(s)\cdot \bigl\vert L _{G}(v) \bigr\vert \cdot \bigl\vert g_{n}(s,v)-1 \bigr\vert \,ds \biggr) \\ & {} + 2\sup_{t\in [0,T]} \bigl\vert I_{3}^{n}(t) \bigr\vert +2 \sup_{ t\in [0, T]} \bigl\vert I^{n}_{5}(t) \bigr\vert . \end{aligned}$$

Then, by Lemma 4.2, (27), (30), and Gronwall’s lemma, we have

$$\begin{aligned} \kappa^{n}(T) \le {}& 2 \Bigl( \sup_{t\in [0,T]} \bigl\vert I_{3}^{n}(t) \bigr\vert + \sup _{ t\in [0, T]} \bigl\vert I^{n}_{5}(t) \bigr\vert \Bigr) \\ & {} \times \exp \biggl\{ C(T) \biggl( \int_{0}^{T} \bigl\vert f_{n}(s) \bigr\vert \,ds+4 \int_{0} ^{T} \bigl\vert L_{G}(v) \bigr\vert \cdot \bigl\vert g_{n}(s,v)-1 \bigr\vert \,ds \biggr) \biggr\} \\ \le{}& C \Bigl( \sup_{t\in [0,T]} \bigl\vert I_{3}^{n}(t) \bigr\vert + \sup_{ t\in [0, T]} \bigl\vert I ^{n}_{5}(t) \bigr\vert \Bigr) \longrightarrow 0. \end{aligned}$$

The proof is complete. □

We now verify the second part of Condition 3.1.

Let $\phi_{\varepsilon }=(\psi_{\varepsilon }, \varphi_{\varepsilon })\in \widetilde{\mathcal{U}}^{\varUpsilon }$ and $\vartheta_{\varepsilon }=\frac{1}{ \varphi_{\varepsilon }}$. The following lemma follows from [8, Lemma 2.3].

### Lemma 4.5

([8, Lemma 2.3])

*The processes*

$$\begin{aligned} \mathcal{E}^{\varepsilon }_{t}(\vartheta_{\varepsilon }) :=& \exp \biggl\{ \int_{(0,t]\times E \times [0,\varepsilon ^{-1}\varphi_{\varepsilon }]}\log\bigl(\vartheta _{\varepsilon }(s,x)\bigr) \overline{N}(\,ds\,dx\,dr) \\ &{} + \int_{(0,t]\times E\times [0,\varepsilon ^{-1}\varphi_{\varepsilon }]}\bigl(-\vartheta_{\varepsilon }(s,x)+1\bigr)\overline{ \vartheta }_{T}(\,ds\,dx\,dr) \biggr\} \end{aligned}$$

*and*

$$ \widetilde{\mathcal{E}}^{\varepsilon }_{t}(\psi_{\varepsilon }) :=\exp \biggl\{ \frac{1}{ \sqrt{\varepsilon }} \int_{0}^{t}\psi_{\varepsilon }(s)\,dB(s)- \frac{1}{2\varepsilon } \int_{0} ^{t} \bigl\vert \psi_{\varepsilon }(s) \bigr\vert ^{2}\,ds \biggr\} $$

*are* $\{\bar{\mathcal{F}}_{t}^{\bar{\mathbb{V}}}\}$-*martingales*. *Set*

$$ \bar{\mathcal{E}}^{\varepsilon }_{t}(\psi_{\varepsilon }, \vartheta_{\varepsilon }):= \widetilde{\mathcal{E}}^{\varepsilon }_{t}( \psi_{\varepsilon })\cdot \mathcal{E}^{\varepsilon } _{t}( \vartheta_{\varepsilon }). $$

*Then*

$$ \mathbb{Q}^{\varepsilon }_{t}(G):= \int_{G}\bar{\mathcal{E}}^{\varepsilon }_{t}( \psi_{\varepsilon }, \vartheta_{\varepsilon })\,d\bar{\mathbb{P}}^{\bar{\mathbb{V}}}, \quad \textit{for } G\in \mathcal{B}(\bar{\mathbb{V}}) $$

*defines a probability measure on* $\bar{\mathbb{V}}$.

Since $( \sqrt{\varepsilon }B+\int_{0}^{\cdot }\psi_{\varepsilon }(s)\,ds,\varepsilon N^{\varepsilon ^{-1}\varphi_{\varepsilon }} ) $ under $\mathbb{Q}^{\varepsilon }_{T}$ has the same law as that of $( \sqrt{\varepsilon }B,\varepsilon N^{\varepsilon ^{-1}} ) $ under $\overline{\mathbb{P}}^{\overline{\mathbb{V}}}$, there exists a unique solution to the following controlled stochastic evolution equation $\widetilde{X}^{\varepsilon }$:

33 $$\begin{aligned} d \widetilde{X}^{\varepsilon }_{t}= {} &b \bigl( \widetilde{X}^{\varepsilon }_{t} \bigr) \,dt+ \sqrt{\varepsilon }\sigma \bigl( \tilde{X}^{\varepsilon }_{t} \bigr) \,dB(t)+\sigma \bigl( \tilde{X} ^{\varepsilon }_{t} \bigr) \psi_{\varepsilon }(t)\,dt \\ & {} + \int_{E} F \bigl( \widetilde{X}^{\varepsilon }_{t-}, z \bigr) \bigl( \varepsilon N^{\varepsilon ^{-1}\varphi_{\varepsilon }}(\,dt\,dz)-\vartheta (dz)\,dt \bigr) + \widetilde{K}_{t} ^{\varepsilon } \\ = {} &b \bigl( \widetilde{X}^{\varepsilon }_{t} \bigr) \,dt+\sqrt{\varepsilon } \sigma \bigl( \widetilde{X} ^{\varepsilon }_{t} \bigr) \,dB(t)+\sigma \bigl( \widetilde{X}^{\varepsilon }_{t} \bigr) \psi_{\varepsilon }(t)\,dt \\ & {} + \int_{E} F \bigl( \widetilde{X}^{\varepsilon }_{t-}, v \bigr) \cdot \bigl( \varphi_{\varepsilon }(t,v)-1 \bigr) \vartheta (dv)\,dt \\ & {} +\varepsilon \int_{E} F \bigl( \widetilde{X}^{\varepsilon }_{t-}, v \bigr) \bigl( N^{\varepsilon ^{-1}\varphi_{\varepsilon }}(\,dt\,dv)-\varepsilon ^{-1} \varphi_{\varepsilon }(t,v)\vartheta (dv)\,dt \bigr) +d \widetilde{K}_{t}^{\varepsilon }. \end{aligned}$$

Here $\widetilde{K}_{t}^{\varepsilon }$ is given by

34 $$\begin{aligned} \widetilde{K}_{t}^{\varepsilon }=\sup _{s\le t}\inf \bigl\{ x\ge 0, \mathbb {E}\bigl[h\bigl(x+ \widetilde{U}_{s}^{\varepsilon }\bigr)\bigr]\ge 0 \bigr\} \end{aligned}$$

with the process $(\widetilde{U}_{t}^{\varepsilon })_{0\le t\le T}$ defined by

35 $$\begin{aligned} \widetilde{U}_{t}^{\varepsilon }= {} & X_{0}+ \int_{0}^{t} b \bigl( \widetilde{X}^{\varepsilon } _{s} \bigr) \,ds+\sqrt{\varepsilon } \int_{0}^{t} \sigma \bigl( \tilde{X}^{\varepsilon } _{s} \bigr) \,dB(s)+ \int_{0}^{t}\sigma \bigl( \tilde{X}^{\varepsilon }_{s} \bigr) \psi_{\varepsilon }(s)\,ds \\ & {} + \int_{0}^{t} \int_{E} F \bigl( \widetilde{X}^{\varepsilon }_{s-}, z \bigr) \bigl( \varepsilon N^{\varepsilon ^{-1}\varphi_{\varepsilon }}(\,ds\,dz)-\vartheta (dz)\,ds \bigr) . \end{aligned}$$

Then

36 $$\begin{aligned} \mathcal{G}^{\varepsilon } \biggl( \sqrt{\varepsilon }B+ \int_{0}^{\cdot }\psi_{\varepsilon }(s)\,ds,\varepsilon N^{\varepsilon ^{-1}\varphi_{\varepsilon }} \biggr) =\widetilde{X}^{\varepsilon }. \end{aligned}$$

The following estimate can be proved in a similar way to (4), which is omitted here.

### Lemma 4.6

*There exists some constant* $\varepsilon _{0}>0$ *such that*

$$ \sup_{0< \varepsilon \le \varepsilon _{0}}\mathbb {E}\Bigl[ \sup_{0\le t\le T} \bigl\vert \widetilde{X} ^{\varepsilon }_{t} \bigr\vert ^{2} \Bigr] < +\infty . $$

Let $\{Y_{\varepsilon }\}_{\varepsilon \in (0,1)}$ be a sequence of random elements of $D([0,T];\mathbb{R})$, and $\{\tau_{\varepsilon },\delta_{\varepsilon }\}$ be such that:

- For each *ε*, $\tau_{\varepsilon }$ is a stopping time with respect to the natural *σ*-field and takes only finitely many values.
- The constant $\delta_{\varepsilon }\in [0,T]$ satisfies that $\delta_{\varepsilon } \rightarrow 0$ as $\varepsilon \rightarrow 0$.

We introduce the following condition on $\{Y_{\varepsilon }\}$:

### Condition (A)

*For each sequence* $\{\tau_{\varepsilon },\delta_{\varepsilon }\}$ *satisfying* (a) *and* (b), $Y_{\varepsilon }(\tau_{\varepsilon }+\delta_{\varepsilon })-Y_{\varepsilon }( \tau_{\varepsilon })\rightarrow 0$ *in probability*, *as* $\varepsilon \rightarrow 0$. *Recall the following lemma from Aldous* [1].

### Lemma 4.7

([1])

*Suppose that* $\{Y_{\varepsilon }\}_{\varepsilon \in (0,1)}$ *satisfies Condition *(A) *and* $\{Y_{\varepsilon }(t)\}_{\varepsilon \in (0,1)}$ *is tight on* $\mathbb {R}$ *for each* $t\in [0,T]$, *then* $\{Y_{\varepsilon }\}_{\varepsilon \in (0,1)}$ *is tight in* $D([0,T];\mathbb{R})$.

### Proposition 4.8

*Fix* ${\varUpsilon }\in \mathbb{N}$, *and let* $\phi_{\varepsilon }=( \psi_{\varepsilon },\varphi_{\varepsilon })$, $\phi =(\psi ,\varphi ) \in \widetilde{\mathcal{U}}^{\varUpsilon }$ *be such that* $\phi_{\varepsilon }$ *converges in distribution to* $\phi =(\psi , \varphi )$ *as* $\varepsilon \rightarrow 0$. *Then*

$$ \mathcal{G}^{\varepsilon } \biggl( \sqrt{\varepsilon }B+ \int_{0}^{ \cdot }\psi_{\varepsilon }(s)\,ds, \varepsilon N^{\varepsilon^{-1} \varphi_{\varepsilon }} \biggr) \Longrightarrow \mathcal{G}^{0} \biggl( \int _{0}^{\cdot }\psi (s)\,ds, \vartheta^{\varphi } \biggr) . $$

### Proof

First, we prove that $\widetilde{X}^{\varepsilon }=\mathcal{G}^{\varepsilon } ( \sqrt{\varepsilon }\beta +\int_{0}^{\cdot }\psi_{\varepsilon }(s)\,ds, \varepsilon N^{\varepsilon^{-1}\varphi_{\varepsilon }} ) $ is tight in $D([0,T];\mathbb {R})$. With the help of Aldous’ criterion in Lemma 4.7, we only need to check that $\widetilde{X}^{\varepsilon }$ satisfies the condition of Lemma 4.7.

By Lemma 4.6,

$$ \sup_{0< \varepsilon < \varepsilon _{0}}\mathbb {P}\bigl( \bigl\vert \widetilde{X}^{\varepsilon }_{t} \bigr\vert >L \bigr) \le C/L^{2}. $$

Hence $\{\widetilde{X}^{\varepsilon }_{t}\}$ is tight on $\mathbb {R}$ for each $t\in [0,T]$. Thus it remains to prove that $\{ \widetilde{X}^{\varepsilon } _{t} \} $ satisfies Condition (A). For any sequences $\{\tau_{\varepsilon }, \delta_{\varepsilon }\}$ satisfying (a) and (b),

37 $$\begin{aligned} \widetilde{X}^{\varepsilon }_{\tau_{\varepsilon }+\delta_{\varepsilon }}-\widetilde{X}^{\varepsilon }_{ \tau_{\varepsilon }}= {} & \int_{\tau_{\varepsilon }}^{\tau_{\varepsilon }+\delta_{\varepsilon }} b \bigl( \widetilde{X} ^{\varepsilon }_{s} \bigr) \,ds+\sqrt{\varepsilon } \int_{\tau_{\varepsilon }}^{\tau_{\varepsilon }+\delta _{\varepsilon }} \sigma \bigl( \widetilde{X}^{\varepsilon }_{s} \bigr) \,d\beta (s)+ \int_{\tau_{\varepsilon }}^{\tau_{\varepsilon }+\delta_{\varepsilon }}\sigma \bigl( \widetilde{X} ^{\varepsilon }_{s} \bigr) \psi_{\varepsilon }(s)\,ds \\ & {} + \int_{\tau_{\varepsilon }}^{\tau_{\varepsilon }+\delta_{\varepsilon }} \int_{E} F \bigl( \widetilde{X} ^{\varepsilon }_{s},v \bigr) \bigl( \varphi_{\varepsilon }(s,v)-1 \bigr) \vartheta (dv)\,ds \\ & {} + \int_{\tau_{\varepsilon }}^{\tau_{\varepsilon }+\delta_{\varepsilon }} \int_{E} \varepsilon F \bigl( \widetilde{X} ^{\varepsilon }_{s}, v \bigr) \bigl( N^{\varepsilon ^{-1}\varphi_{\varepsilon }}(\,ds\,dv)-\varepsilon ^{-1} \varphi_{\varepsilon }(s,v)\vartheta (dv)\,ds \bigr) \\ & {} + \bigl( \widetilde{K}^{\varepsilon }_{\tau_{\varepsilon }+\delta_{\varepsilon }}-\widetilde{K} ^{\varepsilon }_{\tau_{\varepsilon }} \bigr) \\ =:{} &\mathit {II}_{1}^{\varepsilon }+\mathit {II}_{2}^{\varepsilon }+\mathit {II}_{3}^{\varepsilon }+\mathit {II}_{4}^{\varepsilon }+\mathit {II}_{5}^{\varepsilon }+\mathit {II}_{6}^{\varepsilon }. \end{aligned}$$

By the linear growth of *b* and *σ*, we have

38 $$\begin{aligned}& \begin{aligned}[b] \mathbb {E}\bigl[ \bigl\vert \mathit {II}_{1}^{\varepsilon } \bigr\vert \bigr] \le {}& \mathbb {E}\biggl[ \int_{\tau_{\varepsilon }}^{ \tau_{\varepsilon }+\delta_{\varepsilon }} \bigl\vert b \bigl( \widetilde{X}^{\varepsilon }_{s} \bigr) \bigr\vert \,ds \biggr] \\ \le {}& \delta_{\varepsilon }K^{\frac{1}{2}}\mathbb {E}\Bigl[ 1+2 \sup _{0\le t\le T} \bigl\vert \widetilde{X}^{\varepsilon }_{t} \bigr\vert ^{2} \Bigr] ^{\frac{1}{2}} \\ \le {}& \delta_{\varepsilon }K^{\frac{1}{2}} \Bigl( 1+2\mathbb {E}\Bigl[ \sup _{0\le t \le T} \bigl\vert \widetilde{X}^{\varepsilon }_{t} \bigr\vert ^{2} \Bigr] \Bigr) ^{ \frac{1}{2}},\end{aligned} \end{aligned}$$

39 $$\begin{aligned}& \begin{aligned}[b] \mathbb {E}\bigl[ \bigl\vert \mathit {II}_{2}^{\varepsilon } \bigr\vert ^{2} \bigr] \le {}& \varepsilon \mathbb {E}\biggl[ \int_{\tau_{\varepsilon }} ^{\tau_{\varepsilon }+\delta_{\varepsilon }} \bigl\vert \sigma \bigl( \widetilde{X}^{\varepsilon }_{s} \bigr) \bigr\vert ^{2}\,ds \biggr] \\ \le {}& \varepsilon \mathbb {E}\biggl[ \int_{\tau_{\varepsilon }}^{\tau_{\varepsilon }+\delta_{\varepsilon }} K \Bigl( 1+2 \sup _{0\le t\le T} \bigl\vert \widetilde{X}^{\varepsilon }_{t} \bigr\vert ^{2} \Bigr) \,ds \biggr] \\ \le {}& \varepsilon \delta_{\varepsilon }K \Bigl( 1+2\mathbb {E}\Bigl[ \sup_{0\le t\le T} \bigl\vert \widetilde{X} ^{\varepsilon }_{t} \bigr\vert ^{2} \Bigr] \Bigr) ,\end{aligned} \end{aligned}$$

and

40 $$\begin{aligned} \mathbb {E}\bigl[ \bigl\vert \mathit {II}_{3}^{\varepsilon } \bigr\vert \bigr]\le {}& \mathbb {E}\biggl[ \int_{\tau_{\varepsilon }}^{\tau_{\varepsilon }+ \delta_{\varepsilon }} \bigl\vert \sigma \bigl( \widetilde{X}^{\varepsilon }_{s} \bigr) \bigr\vert \cdot \bigl\vert \psi_{\varepsilon }(s) \bigr\vert \,ds \biggr] \\ \le {}& \mathbb {E}\biggl[ \int_{\tau_{\varepsilon }}^{\tau_{\varepsilon }+\delta_{\varepsilon }} K^{ \frac{1}{2}}\mathbb {E}\Bigl[ 1+2 \sup _{0\le t\le T} \bigl\vert \widetilde{X}^{\varepsilon } _{t} \bigr\vert ^{2} \Bigr] ^{\frac{1}{2}} \cdot \bigl\vert \psi_{\varepsilon }(s) \bigr\vert \,ds \biggr] \\ \le {}& K^{\frac{1}{2}} \biggl( \mathbb {E}\biggl[ \int_{\tau_{\varepsilon }}^{\tau_{\varepsilon }+ \delta_{\varepsilon }} \Bigl( 1+2 \sup_{0\le t\le T} \bigl\vert \widetilde{X}^{\varepsilon } _{t} \bigr\vert ^{2} \Bigr) \,ds \biggr] \biggr) ^{\frac{1}{2}}\cdot \biggl( \mathbb {E}\int_{\tau_{\varepsilon }}^{\tau_{\varepsilon }+\delta_{\varepsilon }} \bigl\vert \psi_{\varepsilon }(s) \bigr\vert ^{2}\,ds \biggr) ^{\frac{1}{2}} \\ \le {}& K^{\frac{1}{2}}\delta_{\varepsilon }^{\frac{1}{2}} \Bigl( \mathbb {E}\Bigl[ 1+2 \sup_{0\le t\le T} \bigl\vert \widetilde{X}^{\varepsilon }_{t} \bigr\vert ^{2} \Bigr] \Bigr) ^{\frac{1}{2}}\cdot \biggl( \mathbb {E}\biggl[ \int_{0}^{T} \bigl\vert \psi_{\varepsilon }(s) \bigr\vert ^{2}\,ds \biggr] \biggr) ^{\frac{1}{2}} \\ \le {}& \sqrt{2\varUpsilon }\delta_{\varepsilon }^{\frac{1}{2}} K^{\frac{1}{2}} \Bigl( 1+2\mathbb {E}\Bigl[ \sup_{0\le t\le T} \bigl\vert \widetilde{X}^{\varepsilon }_{t} \bigr\vert ^{2} \Bigr] \Bigr) ^{\frac{1}{2}}. \end{aligned}$$

For any $\eta >0$, when $\delta_{\varepsilon }<\delta $, where *δ* is the constant in Lemma 4.2(ii), we have, by Lemma 4.2,

41 $$\begin{aligned} \mathbb {E}\bigl[ \bigl\vert \mathit {II}_{4}^{\varepsilon } \bigr\vert \bigr]\le {}& \mathbb {E}\biggl[ \int_{\tau_{\varepsilon }}^{\tau_{\varepsilon }+ \delta_{\varepsilon }} \int_{E} \Bigl( 1+2 \sup_{0\le t\le T} \bigl\vert \widetilde{X} ^{\varepsilon }_{t} \bigr\vert \Bigr) \cdot \bigl\vert M_{F}( v) \bigr\vert \cdot \bigl\vert \varphi_{\varepsilon }(s,v)-1 \bigr\vert \vartheta (dv)\,ds \biggr] \\ \le {} & \mathbb {E}\Bigl[ 1+2 \sup_{0\le t\le T} \bigl\vert \widetilde{X}^{\varepsilon }_{t} \bigr\vert \Bigr] \cdot \biggl[ \int_{\tau_{\varepsilon }}^{\tau_{\varepsilon }+\delta_{\varepsilon }} \int_{E} \bigl\vert M _{F}(v) \bigr\vert \cdot \bigl\vert \varphi_{\varepsilon }(s,v)-1 \bigr\vert \vartheta (dv)\,ds \biggr] \\ \le {} & \eta \mathbb {E}\Bigl[ 1+2 \sup_{0\le t\le T} \bigl\vert \widetilde{X}^{\varepsilon } _{t} \bigr\vert \Bigr] . \end{aligned}$$

For the fifth term,

42 $$\begin{aligned} \mathbb {E}\bigl[ \bigl\vert \mathit {II}_{5}^{\varepsilon } \bigr\vert ^{2}\bigr]\le {} & \varepsilon \mathbb {E}\biggl[ \int_{\tau_{\varepsilon }}^{\tau _{\varepsilon }+\delta_{\varepsilon }} \int_{E} \bigl\vert F \bigl( \widetilde{X}^{\varepsilon }_{s}, v \bigr) \bigr\vert ^{2}\cdot \varphi_{\varepsilon }(s,v)\vartheta (dv)\,ds \biggr] \\ \le {} & \varepsilon \Bigl( 1+2 \mathbb {E}\Bigl[ \sup_{0\le t\le T} \bigl\vert \widetilde{X} ^{\varepsilon }_{t} \bigr\vert \Bigr] \Bigr) \sup _{{g\in S^{\varUpsilon }}} \int_{0} ^{T} \int_{E} \bigl\vert M_{F}(v) \bigr\vert ^{2}\cdot g(s,v)\vartheta (dv)\,ds. \end{aligned}$$

According to Lemma 4.1, we know that

43 $$\begin{aligned} \bigl\vert K_{t}^{q_{n}}-K^{q}_{t} \bigr\vert \le {} & \frac{M}{m}\sup_{0\le s \le t}\mathbb {E}\bigl[ \bigl\vert \widetilde{U}^{\varepsilon }_{s}-\widetilde{U}^{\varepsilon }_{s} \bigr\vert \bigr] = \frac{M}{m}\sup_{0\le s\le t}\sum _{i=1}^{5} \mathbb {E}\bigl[ \bigl\vert I^{n}_{i}(s) \bigr\vert \bigr]. \end{aligned}$$

By (37)–(43), Lemma 4.2, and Chebyshev’s inequality, we obtain Condition (A). Thus we have proved that $\widetilde{X}^{\varepsilon }= \mathcal{G}^{\varepsilon } ( \sqrt{\varepsilon }B+\int_{0}^{ \cdot }\psi_{\varepsilon }(s)\,ds, \varepsilon N^{\varepsilon^{-1} \varphi_{\varepsilon }} ) $ is tight in $D([0,T];\mathbb {R})$.

Finally, we prove that $\mathcal{G}^{0} ( \int_{0}^{\cdot }\psi (s)\,ds, \vartheta^{\varphi } ) $ is the unique limit of $\mathcal{G}^{\varepsilon } (\sqrt{ \varepsilon }B+\int_{0}^{\cdot }\psi_{\varepsilon }(s)\,ds, \varepsilon N^{\varepsilon^{-1}\varphi_{\varepsilon }} )$.

Recall (33). Denote

$$ \overline{M}^{\varepsilon }(t):=\sqrt{\varepsilon } \int_{0}^{t} \sigma \bigl( \widetilde{X} ^{\varepsilon }_{s} \bigr) \,dB(s) $$

and

$$ M^{\varepsilon }:= \int_{0}^{t} \int_{E} \varepsilon F \bigl( \widetilde{X}^{\varepsilon }_{s}, v \bigr) \bigl( N^{\varepsilon ^{-1}\varphi_{\varepsilon }}(\,ds\,dv)-\varepsilon ^{-1} \varphi_{\varepsilon }(s,v) \vartheta (dv)\,ds \bigr) . $$

Similar to the proof of (39), we know that $\overline{M} ^{\varepsilon }\Longrightarrow 0$ and $M^{\varepsilon }\Longrightarrow 0$ as $\varepsilon \rightarrow 0$.

Choose a subsequence along which $(\widetilde{X}^{\varepsilon }, \phi_{\varepsilon }, \overline{M}^{\varepsilon }, M^{\varepsilon })$ converges to $(\widetilde{X}, \phi , 0,0)$ in distribution. By the Skorokhod representation theorem, we may assume that $(\widetilde{X}^{\varepsilon }, \phi_{\varepsilon }, \overline{M}^{\varepsilon }, M^{\varepsilon })$ converges to $(\widetilde{X}, \phi , 0,0)$ almost surely.

Note that convergence in Skorokhod topology to a continuous limit is equivalent to the uniform convergence, and $C([0,T];\mathbb {R})$ is a closed subset of $D([0,T];\mathbb {R})$. Hence

$$ \lim_{\varepsilon \rightarrow 0}\sup_{s\in [0,T]} \bigl\vert M^{\varepsilon } \bigr\vert ^{2}=0,\quad \mathbb {P}\text{-a.s.} $$

Since $\widetilde{X}^{\varepsilon }-M^{\varepsilon }\in C([0,T];\mathbb {R})$ and $\widetilde{X}^{\varepsilon }-M^{\varepsilon }\rightarrow \widetilde{X}$ almost surely in $D([0,T];\mathbb {R})$, we have $\widetilde{X}\in C([0,T];\mathbb {R})$, and

$$ \lim_{\varepsilon \rightarrow 0}\sup_{s\in [0,T]} \bigl\vert \widetilde{X}^{\varepsilon }(s)- \widetilde{X}(s) \bigr\vert =0,\quad \mathbb {P}\text{-a.s.} $$

Letting $\varepsilon \rightarrow 0$, along the lines of the proof of Proposition 4.4, we see that *X̃* must solve

44 $$ \textstyle\begin{cases} \widetilde{X}_{t}=X_{0}+\int_{0}^{t} b(\widetilde{X}_{s-})\,ds+\int_{0} ^{t} \sigma (\widetilde{X}_{s-})\psi \,ds \\ \hphantom{\widetilde{X}_{t}=}{}+\int_{0}^{t}\int_{E} F( \widetilde{X}_{s-}, v) ( \varphi (s,v)-1 ) \vartheta (dv)\,ds+ \widetilde{K}_{t}, \\ \mathbb {E}[h(\widetilde{X}_{t})]\ge 0, \quad\quad \int_{0}^{t} \mathbb {E}[h(\widetilde{X}_{s})]\,d\widetilde{K}_{s}=0, \quad t \ge 0. \end{cases} $$

By the uniqueness, this gives that $\widetilde{X}=\mathcal{G}^{0} ( \int_{0}^{\cdot }\psi (s)\,ds, \vartheta^{\varphi } ) $.

The proof is complete. □
